# Non-Invasive Brain Stimulation for the Treatment of Symptoms Following Traumatic Brain Injury

**DOI:** 10.3389/fpsyt.2015.00119

**Published:** 2015-08-26

**Authors:** Simarjot K. Dhaliwal, Benjamin P. Meek, Mandana M. Modirrousta

**Affiliations:** ^1^Department of Psychiatry, University of Manitoba, Winnipeg, MB, Canada

**Keywords:** traumatic brain injury, non-invasive brain stimulation, repetitive transcranial magnetic stimulation, trancranial direct current stimulation, rehabilitation, depression, altered states of consciousness

## Abstract

**Background:**

Traumatic brain injury (TBI) is a common cause of physical, psychological, and cognitive impairment, but many current treatments for TBI are ineffective or produce adverse side effects. Non-invasive methods of brain stimulation could help ameliorate some common trauma-induced symptoms.

**Objective:**

This review summarizes instances in which repetitive Transcranial Magnetic Stimulation (rTMS) and transcranial Direct Current Stimulation (tDCS) have been used to treat symptoms following a TBI. A subsequent discussion attempts to determine the value of these methods in light of their potential risks.

**Methods:**

The research databases of PubMed/MEDLINE and PsycINFO were electronically searched using terms relevant to the use of rTMS and tDCS as a tool to decrease symptoms in the context of rehabilitation post-TBI.

**Results:**

Eight case-studies and four multi-subject reports using rTMS and six multi-­subject studies using tDCS were found. Two instances of seizure are discussed.

**Conclusion:**

There is evidence that rTMS can be an effective treatment option for some post-TBI symptoms, such as depression, tinnitus, and neglect. Although the safety of this method remains uncertain, the use of rTMS in cases of mild TBI without obvious structural damage may be justified. Evidence on the effectiveness of tDCS is mixed, highlighting the need for additional investigations.

## Introduction

### Traumatic brain injury

Traumatic brain injury (TBI) involves the temporary or permanent impairment of brain function following physical trauma to the head. TBI commonly occurs as result of falls, motor vehicle accidents, combat trauma, intentional violence, and sports-related incidents (1). TBI is a leading cause of death and disability, particularly amongst the young and the elderly (1). The annual incidence of TBI in North America is ~2000 out of every 1 million individuals, with an estimated 1.5 million Americans experiencing a TBI each year (1, 2).

Physical damage to brain tissue and blood vessels can occur directly as a result of a focal impact to the skull or a rapid acceleration of the head. However, brain damage is not limited to the moment of initial trauma, as subsequent intracranial events can also cause a great deal of problems. These secondary injuries can result from both physical and biochemical processes in the brain, such as glutamatergic excitotoxicity, a compromised blood brain barrier, disrupted blood vessels, and changes in intracranial pressure that can lead to contusions, hemorrhages, edemas, or hematomas. Furthermore, tissue damage is not always limited to the initial point of skull impact. Damage can also occur on the opposite side of the brain to the point of impact – contra-coup – or more diffusely, with frontal and temporal lobes often bearing the brunt of such disturbance (1).

Brain injury occurs along a continuum of severity, affecting a variety of structures depending on the location of impact. Thus, despite many individuals exhibiting ostensibly similar injuries, there is a large heterogeneity in the presentation of symptoms (3). Impairments following a mild TBI can be affective (depression, anxiety, and psychosis), somatic (tinnitus, hypersensitivity to noise and light), and/or cognitive (deficits in attention, concentration, information processing, memory, problem solving, and thinking) (3–6). Affective symptoms are common following TBI, with anywhere from 10 to 77% of patients experiencing the onset of depressive symptoms post-injury (6–8). Somatic complaints are also frequent, with tinnitus being reported in roughly 50% of TBI cases and some individuals experiencing auditory hallucinosis (9). Severe TBI involves more extensive trauma, with injuries often leading to permanent impairment of brain functions and disorders of consciousness, ranging from comatose to minimally conscious (10).

The current treatments available for rehabilitation following a TBI vary greatly depending on the primary symptoms being expressed. Pharmacotherapy is a common treatment for TBI-induced depression, but reports are mixed as to its effectiveness. Furthermore, due to brain vulnerability following a TBI, the side effects of psychotropic medications are often accentuated. Tricyclic antidepressants, for example, have been reported to have adverse effects, including seizures, in patients with TBI (6). In a 2009 review, Fann and colleagues found no definitive evidence for the efficacy of any specific class of medications in the treatment of depression in TBI (6). The authors highlight preliminary evidence for SSRIs – particularly sertraline (11, 12) and citalopram (13) – and one dual-action SNRI [milnacipran (14)] as potentially useful interventions, as well as reports that methylphenidate may improve the rate of functional recovery in the post-acute phase of TBI (15, 16). However, evidence for the efficacy of these interventions is not always consistent (17), and the use of other agents, such as bromocriptine, amantadine, donepezil, levodopa/carbidopa, and dextroamphetamine, are largely limited to case reports (18). At present, no medication has received approval from the United States Food and Drug Administration (FDA) for the treatment of any neuropsychiatric consequence of TBI. Many different methods have been employed to reduce the severity of TBI-induced tinnitus and auditory hallucinations including anti-anxiety medication, acupuncture, osteopathy, hyperbaric oxygen therapy, steroids, acoustic stimulation, and behavioral intervention with varying degrees of success (19–21). In cases of severe TBI, standard rehabilitation attempts to improve functional recovery have generally displayed limited utility (22).

These examples highlight the need for additional therapies to be explored for effective management of symptoms following TBI. To this end, non-invasive neurostimulatory and neuromodulatory tools, such as transcranial Direct Current Stimulation (tDCS) and repetitive Transcranial Magnetic Stimulation (rTMS), may offer therapeutic alternatives for the treatment of symptoms and sequelae following TBI.

### Transcranial magnetic stimulation

Transcranial magnetic stimulation is a neuromodulatory tool used to non-invasively induce neural activity through the use of rapidly alternating magnetic fields. A TMS machine contains a capacitor that produces a brief electrical current which is run through a coil, creating a magnetic field centered at the focal point of the coil. The magnetic field is able to pass through the bone of the skull and induce activity in cortical neurons below. Pulses can be delivered repetitively to produce long-term changes in neural activity. Cortical excitability can be either increased or decreased through the application of high- (>5 Hz) or low- (1 Hz) frequency stimulation, respectively (23, 24). Repetitive TMS (rTMS) therapy has proven to be a safe and effective option for alleviating symptoms of treatment-resistant depression (25–27).

One of the major advantages of rTMS is its relative safety and the absence of major adverse side effects when established guidelines are followed. The most common side effects of rTMS include headache and minor scalp irritation following therapy, but these effects tend to be transient and easily treated with common analgesics (23). More distressing side effects, such as confusion and memory loss, which are commonly associated with electroconvulsive therapy, have not been reported following rTMS. The major concern with rTMS is the risk of seizure-induction (24). Although the incidence of seizure following rTMS in normal populations is low, it is of greater concern when rTMS is being considered for individuals who demonstrate an increased likelihood of seizure. For this reason, conditions that accompany an increased likelihood of seizure, such as TBI, pro-epileptogenic medication, substance abuse, or a family history of epilepsy, are commonly considered contraindications for treatment (23).

Despite the potential risks, past reviews have suggested that insights gained from the therapeutic use of rTMS in patients with non-trauma-induced brain injuries (e.g., stroke, Parkinson’s disease, or spinal cord injury) may be applicable to patients with TBI (22, 28). For example, inhibition of the unaffected hemisphere through the use of low-frequency (1 Hz) rTMS has produced improvements in hand motor dysfunction and aphasic symptoms following stroke (29, 30). Transient inhibition of the contralesional parietal region via low-frequency rTMS has been shown to improve line bisection and clock drawing task performance in patients with visuospatial neglect (31). High-frequency (5 Hz) rTMS applied to the primary motor cortex in the hemisphere contralateral to pain has reduced neuropathic pain and improved clinical spasticity in patients with non-trauma induced brain damage (32–34). The use of rTMS in healthy populations following the induction of virtual lesions also offers insights into the use of rTMS in patients with real brain damage. For example, healthy patients demonstrate riskier decision making when virtual lesions in the right dorsolateral prefrontal cortex (DLPFC) are induced via TMS (35). Based on this observation, Demirtas-Tatlidede et al. suggest that impaired decision making following a TBI may be improved by increasing right DLPFC activity via high-frequency rTMS (22).

Despite the demonstrated utility of single-pulse TMS in the diagnosis of TBI and the potential usefulness of rTMS in treating common TBI symptoms, brain injury is usually considered a contraindication to the repetitive form of TMS due to increased overall neural excitability and seizure risk. For this reason, TBI patients are excluded from most rTMS studies making it difficult to readily assess the efficacy and safety of rTMS as a treatment for TBI (23).

### Transcranial direct current stimulation

Transcranial direct current stimulation is a non-invasive neuromodulatory tool which uses low amplitude direct current to alter neuronal firing (22). Transcranial applications of direct current can induce focal, prolonged shifts of cortical excitability using anodal or cathodal stimulation to increase or decrease cortical excitability, respectively (36, 37). Clinically significant improvements have been seen in patients with brain damage due to stroke, with behavioral effects persisting for weeks following tDCS treatment (38, 39). The administration of tDCS has also been found to be safe and effective in the treatment of major depressive disorder (40–42). Adverse effects of tDCS treatment include moderate fatigue (35%), mild headache (11.8%), nausea (2.9%), and a transient itching sensation in area of stimulation (43, 44). According to Nitsche et al., no cases of seizure have been reported in relation to the use of tDCS as a clinical intervention (45).

With the demonstrated effectiveness of tDCS applications in the treatment of depression (46), Demirtas-Tatlidede et al. propose that these applications could find utility for treating patients with traumatic brain injuries (22). The authors theorize that bilateral tDCS to the left and right DLPFC could result in clinically significant reduction of depressive symptoms with onset following TBI. Such suggestions extend insights made in patients with brain damage due to stroke and suggest that left frontotemporal cathodal tDCS could improve naming in patents with non-fluent aphasia due to TBI (47). Other potential applications of tDCS in cases of TBI include bilateral tDCS to the DLPFC to improve decision-making impairments and modulation of sensorimotor cortical activity to suppress central pain (22, 48).

In this systematic review, we present studies that have used rTMS or tDCS in the treatment of symptoms following a TBI to examine the safety and efficacy of indirect cortical stimulation in this patient population, and we consider the potential impact these results may have on established safety guidelines. Due to the diverse and debilitating nature of traumatic brain injuries and the limited efficacy of available clinical options, these novel treatments are worth exploring.

## Methods

**Figure 1:**
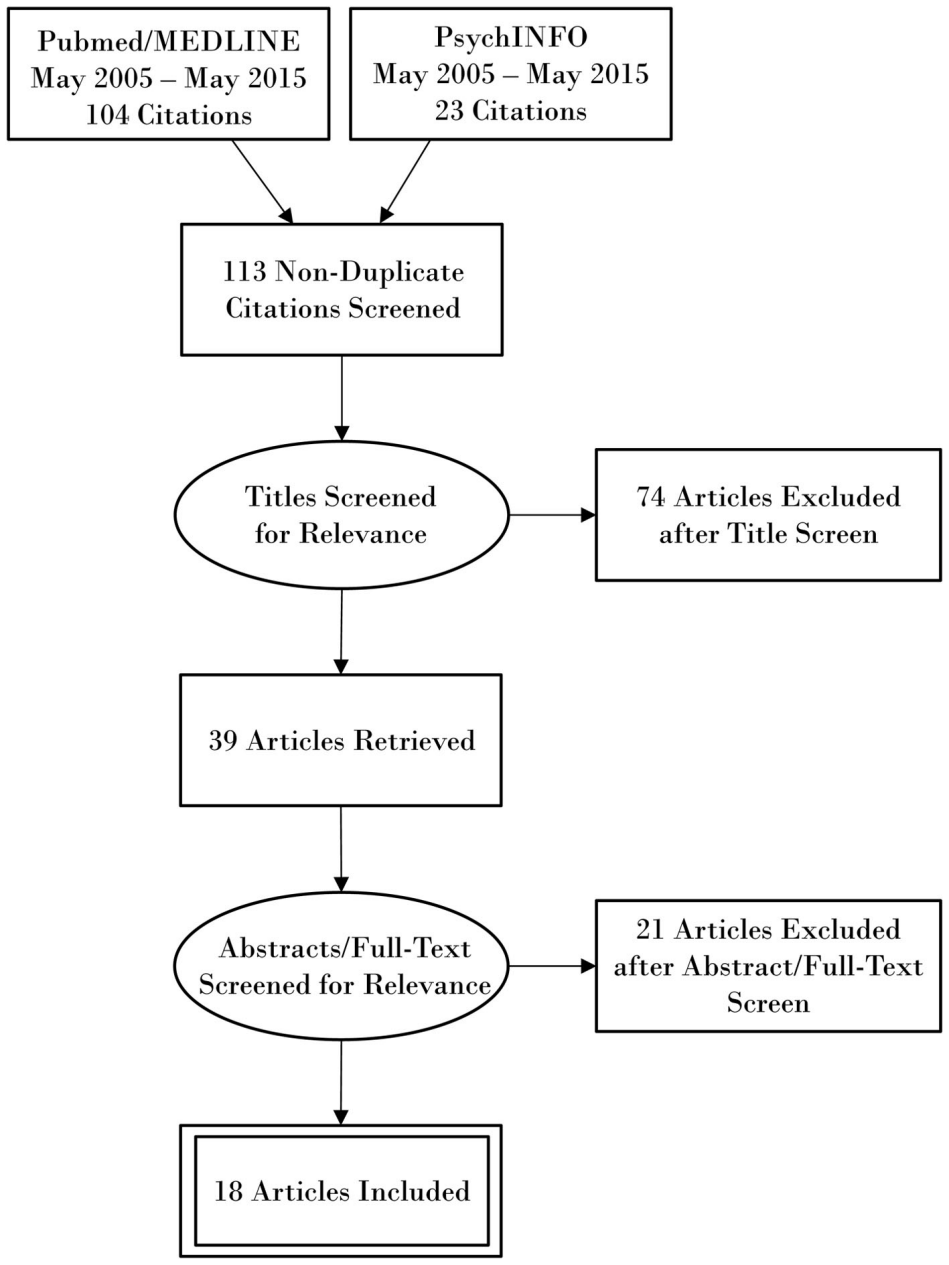
**PRISMA flow diagram outlining search methodology**.

The research databases of PubMed/MEDLINE and PsycINFO were electronically searched using the terms “transcranial magnetic stimulation”, “transcranial direct current stimulation”, “brain injuries”, “concussion”, and “consciousness disorders”. The MeSH approach was utilized to restrict PubMed findings to transcranial magnetic stimulation (TMS) and transcranial direct current stimulation (tDCS) as a tool to decrease symptoms, and to restrict TBI in the context of therapy and rehabilitation. The references of articles found through our search method were also reviewed. Articles were selected manually and limited to English articles from the last 10 years with focus on repetitive TMS (rTMS) and tDCS as tools to decrease symptoms for TBI and rehabilitation post-TBI. A PRISMA flow diagram outlining the search methodology is shown in Figure 1.

## Results

We found 10 articles and 1 abstract that involve the use of rTMS as a therapeutic or rehabilitative tool for symptoms and sequelae following TBI. Three articles involve multiple patients, while the remaining eight manuscripts present single-case reports – two of which describe rTMS-related seizure events. The abstract discusses 11 individuals, only 2 of whom suffered a TBI. These reports address a variety of symptoms, including depression, auditory dysfunction, post-concussion symptoms, hemispatial neglect, and altered states of consciousness. Relevant details from the reviewed rTMS papers are summarized in Table 1. All rTMS studies used figure-eight focal coils to administer pulses unless otherwise noted. We found six articles that describe the use of tDCS in multiple individuals with TBI. Three articles focus on the restoration of cognitive symptoms – primarily attention and memory, two describe attempts to improve altered states of consciousness, and one used tDCS in conjunction with physical therapy to ameliorate upper extremity motor dysfunction. Relevant details from the reviewed tDCS papers are summarized in Table 2. Owing to the heterogeneity of TBI cases, an in-depth examination of the protocols used and any concurrent conditions and/or medications are presented.

**Table 1:** **Summarized details of reviewed rTMS articles**.

Study	Patient descriptions	Brain damage	Target symptoms	Stimulation location	Stimulation parameters	# Sessions	Outcome
Bonni et al. (49)	20 y.o. male; severe TBI 2 years prior	Cortical lesion in right temporal cortex; small cortico–subcortical lesions in periventricular white matter	Hemispatial neglect	Left posterior parietal cortex	50 Hz cTBS at 80% active MT	20 (2/day)	Marked cognitive improvement in BIT
Cavinato et al. (50)	31 y.o. male; severe TBI 8 months prior	Diffuse hematoma in corpus callosum; mass effect over fourth ventricle	Altered consciousness	Left DLPFC	20 Hz at 90% MT	4 prior to seizure	Seizure
Cosentino et al. (20)	63 y.o. male; TBI of unspecified severity 10 months prior	Structural lesion of right temporal lobe	Musical hallucination	Right temporal area	1 Hz at 90% MT using a focal coil	10	Disappearance following Tx, reappearance in reduced state 4 months post-Tx
Fitzgerald et al. (51)	41 y.o. female; mild TBI 14 years prior	None	Depression	DLPFC	Active sequential stimulation at 110% MT: low: 1 Hz; high: 10 Hz	20	Response (50% MADRS reduction)
George et al. (52)	*n* = 41 [*n* = 21 with mTBI (11 sham, 13 active rTMS)]	*Not specified*	Suicidal ideation	Left prefrontal cortex	10 Hz at 110% MT	9 (3/day × 3 days)	Drop in SSI scores not significantly different between groups; trend toward more rapid response in active group
Giovannelli et al. (53)	*n* = 2; Both patients in VS following severe TBI; ages and time since injury not reported	*Not specified*	Altered consciousness	Left primary motor cortex	20 Hz at 60% max output vs sham	5	No significant difference
Koski et al. (54)	*n* = 15 [female = 6; age = 34.3 (10.8) years; PCS score = 37.5 (10.9)] *n(completers)* = 12	*Not specified*	Post-concussion symptoms	Left DLPFC	10 Hz at 110% MT	20	*n* = 9: improvement of symptoms (≥5 reduction on PCS scale); *n* = 1: worsening of symptoms
Kreuzer et al. (19)	53 y.o. male; severe TBI 5 years prior	Frontotemporal epi- and subdural hematoma	Tinnitus	Left primary auditory cortex	1 Hz at 110% resting MT	10 (repeated 5 times in 3 years)	Marked reduction in tinnitus symptoms
Manganotti et al. (55)	*n* = 3	*Patient 1*: subdural hematoma; diffuse cortical lesions	Altered consciousness	Left/right primary motor cortex	20 Hz at 100% MT	1	*Patient 1*: no EEG change, slight increase visual JFK CRS-R
	*Patient 1*: 37 y.o. female in VS 34 months post-TBI	*Patient 2*: multifocal bifrontal lesions					*Patient 2*: no EEG change, slight increase visual JFK CRS-R
	*Patient 2*: 29 y.o. male with MCS 94 months post-TBI	*Patient 3*: pontomesencephalic lesion					*Patient 3*: no difference
	*Patient 3*: 38 y.o. male with MCS 36 months post-TBI						
Pachalska et al. (56)	20 y.o. male; severe TBI 3–4 years prior	Diffuse atrophy and enlarged ventricles in the RH	Impaired executive functioning	Right + left frontal/temporal regions	1 Hz to LH and 5 Hz to RH	20	Large improvements in most tests of executive functioning and all categories of the FBI
Louise-Bender Pape (10)	26 y.o. male in VS 287 days post-TBI	Hemorrhage in lateral and fourth ventricles and right temporal lobe; diffuse subarachnoid hemorrhage	Altered consciousness	Right DLPFC	Paired 100 μs pulse trains with 100 ms ISI and 5 s ITI at 110% MT	30	Increased DOCS; clinical, behavioral improvement
Louise-Bender Pape (57)	32 y.o. male in VS 9 years post-TBI	Multicystic encephalomalacia of RH and basal ganglia; hypoattenuation in the anterior frontal lobe; dilatation of 3rd and lateral ventricles	Altered consciousness	Left DLPFC	Paired 100 μs pulse trains with 100 ms ISI and 5 s ITI at 110% MT	21 prior to seizure; 19 post-seizure	Electrographic seizure; improvement in following one-step commands and purposeful vocalizations prior to seizure

*y.o., years old; TBI, traumatic brain injury; Tx, treatment; cTBS, continuous theta burst stimulation; rTMS, repetitive transcranial magnetic stimulation; tDCS, transcranial direct currant stimulation; DLPFC, dorsolateral prefrontal cortex; MT, motor threshold; BIT, Behavioral Inattention Test; MADRS, Montgomery–Åsberg Depression Rating Scale; SSI, Beck Scale of Suicidal Ideation; PCS, post-concussion syndrome; EEG, electroencephalogram; JFK CRS-C, JFK Coma Recovery Scale-Revised; FBI, frontal behavioral inventory; DOCS, Disorders of Consciousness Scale; RH, right hemisphere; LH, left hemisphere*.

**Table 2:** **Summarized details of reviewed tDCS articles**.

Study	Patient descriptions	Brain damage	Target symptoms	Stimulation location	Stimulation parameters	# Sessions	Outcome
Angelakis et al. (58)	*n* = 4; 3 in PVS, 1 in MCS− following severe closed-head TBI; age range 19–37; 6 months–10 years post-TBI	*Not specified*	Altered consciousness	*Anode*: left DLPFC or left primary sensorimotor cortex	*Week 1*: 1 mA/25 cm^2^ × 20 min	10	*Patient 1*: PVS to MCS− 1 yr post-tDCS; *Patients 2 and 3*: no improvement; *Patient 4*: MCS− to MCS+ 1 week post-tDCS and conscious 2 weeks post-tDCS
				*Cathode*: right orbit	*Week 2*: 2 mA/25 cm^2^ × 20 min	
Kang et al. (59)	*n* = 9, 5 sham; 8 males; age range 20–78; 216.9 (52.5) days post-closed head TBI	*Various* (*all*: SDH or ICH to left or bilateral frontal lobes; *n* = 4: additional parietal, temporal, occipital, or cingulate damage)	Attention deficits	*Anode*: left DLPFC	2 mA/25 cm^2^ × 20 min	1	Immediate but not lasting improvement in attention
				*Cathode*: contralateral supraorbital region			
Les´niak et al. (60)	*n* = 23, 11 sham; age range 20–45 years; 4–92 months post-TBI	*Various* (incl. frontal/parietal/temporal/occipital contusion; cerebellum or BS damage)	Memory and attention deficits	*Anode*: left DLPFC	1 mA/35 cm^−2^ × 10 min	15	No improvements in attention or memory
				*Cathode*: contralateral supraorbital region			
Middleton et al. (61)	*n* = 5 [1 TBI (age 39, time since injury = 206 months), 1 TBI + stroke (age = 38; 9 months post-injury)]	*Not specified*; both TBI patients had motor issues affecting their left UE	Motor impairments	*Anode*: ipsilesional motor cortex	1.5 mA/25 cm^2^ × 15 min	24	Improvement on UE-FM – persisted at a 6-month follow-up
				*Cathode*: contralesional motor cortex			
Thibaut et al. (62)	*n* = 25, 6 post-traumatic UWS; *n* = 30,19 post-traumatic MCS	*Not specified*	Altered consciousness	*Anode*: left DLPFC	2 mA/35 cm^2^ × 20 min	1	*UWS patients*: no improvement; *MCS patients*: improved consciousness
				*Cathode*: contralateral supraorbital region			
Ulam et al. (63)	*n* = 23; 13 treatment [age 31.4 (9.8); 57.4 (37.8) days post-injury], 13 sham [age 35.7 (13.7); 41.1 (20.9) days post-injury]	*Various* (incl. SDH, SAH, frontal/parietal/temporal/occipital contusion; three severe TBI)	Various neuropsych. functions (including attention and memory)	*Anode*: left DLPFC	1 mA/25 cm^2^ × 20 min	10	No difference in neuropsych. improvement between groups *Active group only*: correlation between cortical excitability and neuropsych. improvement
				*Cathode*: contralateral supraorbital region			

*PVS, persistent vegetative state; MCS, minimally conscious state; DLPFC, dorsolateral prefrontal cortex; SDH, subdural hemorrhage; ICH, intracerebral hemorrhage; SAH, subarachnoid hemorrhage; BS, brain stem; UE, upper extremity; UWS, unresponsive wakefulness syndrome; UE-FM, Fugl-Meyer Assessment of Sensorimotor Impairment – upper extremity section*.

## rTMS

### Treatment of post-injury onset of depression and suicidal ideation

Fitzgerald et al. report rTMS treatment for a pharmacotherapy-resistant 41-year-old female patient with severe recurrent depression [Montgomery–Åsberg Depression Rating Scale (MADRS) = 34; Inventory of Depressive Symptomology-Clinical Rated (IDS-CR) = 49] persisting for 14 years with onset following a mild closed-head TBI and no prior history of brain injury (51). Magnetic resonance imaging (MRI) and diffusion tensor imaging (DTI) demonstrated no contraindication to rTMS and no presence of diffuse axonal injuries. Desvenlafaxine was held at a constant dosage 8 weeks prior to treatment and throughout the course of rTMS. Active sequential bilateral rTMS was administered to the DLPFC (right-sided low-frequency rTMS followed by left-sided high-frequency rTMS). A single stimulation train (1 Hz, 900 pulses/session) administered at 110% of resting MT during right-sided low-frequency rTMS was immediately followed by 30 trains (10 Hz, 5 s train duration, 25 s inter-train interval), at 110% of resting MT for left-sided high-frequency rTMS. The patient showed a positive response to treatment with a greater than 50% reduction in depressive symptoms (MADRS = 14; IDS-CR = 21). Neuropsychological assessments of attention, concentration, working memory, speed of information processing, verbal and visual memory, perceptual ability, and executive functioning showed no deleterious changes in cognitive performance. No adverse side effects of treatment were reported.

George et al. administered high-frequency rTMS (10 Hz, 5 s train duration, 10 s IT for 30 min) at 110% resting MT over the left prefrontal cortex to suicidal inpatients (Beck Scale of Suicidal Ideation score ≥12 and ≥3 on Question #3 of the Ham-D) in a randomized, sham-controlled study (52). Each session delivered 6000 pulses over 30 min, and sessions were repeated three times daily with 1 h between sessions for 3 days (total 9 sessions and 54,000 pulses). 41 patients with a diagnosis of either PTSD or mTBI were recruited for this study (20 active and 21 sham). Of these patients, 21 individuals had previously suffered a TBI (13 active and 11 sham). The protocol was generally well tolerated with no major side effects, although one patient suffered a first-degree scalp burn from coil overheating. The authors report a trend toward a more rapid change in SSI in the active rTMS group compared to the sham group, but there was no overall difference in the change in SSI between groups. Patients showed a reduction in how much they were bothered by thoughts of suicide, but they demonstrated no difference in future intent of suicide, thoughts of suicide, self-rated sadness, tiredness, or happiness. The authors note that patients continued to receive standard inpatient care – including changes in medication – throughout the study, so the observed results cannot be separated from any non-specific hospitalization effect. Results were not broken down between patients with PTSD and TBI, so conclusions cannot be drawn regarding the efficacy of this protocol specifically in relation to TBI. However, the fact that this rather aggressive protocol was generally well tolerated demonstrates its feasibility.

### Treatment of post-injury onset of auditory dysfunction

Kreuzer et al. report rTMS treatment of severe tinnitus [tinnitus questionnaire (TQ) = 53] persisting for 4 years following a severe TBI in a complex 53-year-old male patient. The patient presented with co-morbid post-injury onset depression and associated severe sleep disorder, alcohol and benzodiazepine abuse, as well as a single symptomatic seizure immediately following injury with no subsequent seizures (19). Tinnitus symptoms displayed resistance to prior treatments of acupuncture, osteopathy, hyperbaric oxygen therapy, intravenous application of steroids, and acoustic stimulation. Treatment began once alcohol and benzodiazepine intake were excluded via laboratory screening tests administered through the course of rTMS, with antidepressant (75 mg amitriptyline) and antiepileptic (150–300 mg pregabalin) dosage kept constant throughout treatment. Trains of stimulation at 110% resting MT (1 Hz, 2000 stimuli/session) applied to the left primary auditory cortex for 10 sessions demonstrated a positive effect lasting 3 months after the first treatment series (TQ = 38). Four subsequent treatment series over the course of 3 years demonstrated effects lasting 6 months (TQ = 26 after third session, no ratings taken after fourth session). The fifth and final series followed a modified protocol targeted at the right DLPFC (1 Hz, 1000 pulses/session) followed by stimulation applied to the left primary auditory cortex (1 Hz, 1000 pulses/session) for a duration of 5 days (beginning of fifth session TQ = 50; following treatment TQ = 33). The authors state that the patient remained abstinent from alcohol and benzodiazepines throughout treatment. The patient reported a reduction in loudness of tinnitus, and treatment was reported to be well tolerated without adverse effects or seizures as a result of rTMS intervention.

Cosentino et al. report the use of rTMS in the treatment of musical hallucinosis (a form of auditory hallucination) with an onset of 10 months following right temporal injury in a 63-year-old male patient with prior moderate–severe hearing loss (hearing loss remained constant throughout and following treatment) from chronic daily noise exposure over 20 years (20). EEG scans showed an absence of epileptiform abnormalities. PET scans demonstrated hypoactivity in the corresponding temporal lesion and increased metabolic activity in the right posterior temporal lobe. MRI revealed a contusion of the right temporal pole, but neurological and neuropsychological status was normal for age and education level as assessed by memory, attention, language, apraxia, and visuospatial tests. Treatment with antiepileptics (gabapentin and carbamazepine) and antipsychotics (risperidone and paroxetine) was unsuccessful in the months prior to rTMS. Stimulation was applied to the right posterior temporal cortex at 90% MT (1 Hz, 1200 pulses/session; 20 min train duration) for 2 weeks. The severity of musical hallucinations decreased from 5 to 8 points prior to treatment to 2 points post-treatment on a 10-point scale created to measure symptom severity. PET scans during 5 months of follow-up sessions demonstrated decreased hyperactivity of right posterior temporal cortex and decreased metabolic activity of the right posterior temporal lobe but persisting hypoactivity of the temporal lesion. Cognitive abilities were not tested following rTMS intervention. No adverse effects were reported.

### Rehabilitative applications for post-injury cognitive impairments

Theta burst stimulation (short trains of rTMS at high frequencies) applied to the left hemisphere has been seen to improve the symptoms of hemispatial neglect in patients with brain injury due to stroke (64). Bonni et al. extended these insights for use in a patient with brain injury due to trauma (49). The authors used continuous theta burst stimulation (cTBS) to treat severe hemispatial neglect in a 20-year-old male patient with onset following a severe TBI [Behavioral Inattention Test-Conventional (BIT-C) scale ~28] sustained 2 years prior to intervention. Neuropsychological assessments demonstrated mild attentional and executive deficits and minimal memory impairment. Three pulse bursts were applied to the left posterior parietal cortex at 80% active MT (50 Hz, 600 pulses/session; 40 s train interval, 200 ms inter-train interval) twice daily (15 min inter-session interval) for 2 weeks. Functional magnetic resonance images (fMRI) demonstrated decreased excitability of posterior parietal cortex-M1 connections in the left hemisphere and a bilateral increase of functional connectivity in the frontal-parietal network. In conjunction with these functional changes, marked cognitive and clinical improvements were reported and seen to persist 2 weeks following intervention (BIT-C ~ 58). No adverse effects were reported.

Pachalska et al. administered rTMS to a 26-year-old male who had previously (3–5 years) suffered a severe TBI which left him in a coma for 2 months (56). Brain scans revealed diffuse atrophy and enlarged ventricles in the right hemisphere, and the patient exhibited anosognosia, executive dysfunction, behavioral changes; perseverations, fits of uncontrolled laughter, and was sporadically aggressive and impulsive. Twenty sessions of low-frequency (1 Hz) rTMS were administered over left frontal and temporal regions along with high-frequency (5 Hz) rTMS to right frontal and temporal regions. Following rTMS, the patient showed large improvements in most tests of executive functioning, including: general intelligence, attention, visuospatial ability, and logical memory. He also showed improvement on all categories of the frontal behavioral inventory, including: social conduct, personal conduct, mood disorders, and control disorders. The authors state that the patient had previously shown little progress following “traditional rehabilitation” and only small changes following beta training. Although EEG spectra were no different post-rTMS, NO-GO ERP recordings showed improvement compared to pre-rTMS, though still much different from healthy controls. No seizure events or significant adverse side effects were reported.

Recently, Koski et al. examined the effect of high-frequency rTMS on individuals experiencing post-concussion syndrome (PCS) at least 6 months post-injury (54). 15 patients with a score of 22 or greater on the PCS Scale received 20 daily sessions of rTMS (10 Hz at 110% resting MT; 20 5 s trains with 25 s ITI; 1000 pulses/session) over the left DLPFC. Two patients quit due to worsening symptoms and one for unrelated reasons. Of the 12 patients who completed treatment, 9 showed an improvement in symptoms (>5 point decrease on PCS scale) and 1 patient worsened. Patients also showed an overall decrease in ratings of headaches, as well as a (statistically uncorrected) decrease in symptoms of fatigue, trouble falling asleep, difficulty remembering, and numbness or tingling. No seizure events or serious side effects were reported. Nine out of twelve patients found 110% MT stimulation to be intolerable in the first session, but all patients tolerated this intensity by the sixth session following gradual intensity increases. fMRI scans demonstrated greater activations in DLPFC and greater deactivation of the rostral ACC during a working memory task following the last session compared to pre-treatment. Results of a 3-month follow-up with some patients indicate that maintenance treatments may be required to sustain the observed gains.

### Rehabilitative applications for post-injury loss of consciousness

Louise-Bender Pape et al. examined the safety and restorative effects of rTMS by attempting to restore consciousness following a severe TBI (10). They used rTMS to induce neurobehavioral changes in a 26-year-old male who remained in a vegetative state 287 days after a severe TBI. Computerized tomography (CT) scans revealed hemorrhage in the right temporal lobe as well as diffuse traumatic subarachnoid hemorrhage. The patient was described to be in a state of arousal without behavioral evidence of awareness of self or capacity to interact with the environment [Disorders of Consciousness Scale (DOCS) = 50.3]. Prior to treatment, the patient was titrated off neurostimulants (amantadine and methylphenidate) and antispasticity medications and did not receive any neurological medication during the course of the intervention. Safety was ensured by administering a modified lower-than-normal frequency rTMS protocol following completion of a daily medical examination, with an electroencephalogram (EEG) conducted before and after each session and continued bi-weekly for 6 weeks post-treatment. Stimulation was administered to the right DLPFC at 110% abductor pollicis brevis MT (300 trains/session with a 5 s inter-train interval, where each train consisted of a paired-pulse with a 100 ms inter-pulse interval) for 6 weeks. The patient experienced complications that the authors concluded were not related to the rTMS intervention. The patient was treated with 2 g Ceftazidime, 1 g vancomycin, 900 mg amikacin, and 500 mg Cipro. No adverse effects were reported as a result of rTMS treatment, and EEGs performed throughout and following the treatment revealed lack of epileptiform discharges following rTMS. The authors report a change in classification of state of consciousness from vegetative state to minimally conscious (DOCS: 15th session = 58.6; 30th = 53.7; 6 weeks post-treatment = 56.7). Qualitative improvements through the course of rTMS were reported, including markedly improved motor skills and visual ability, the appearance of vocalizations, and the development of basic communication. Continued qualitative improvements were reported a year following rTMS treatment by the patient’s family, with no reported adverse events.

Manganotti and colleagues administered high-frequency rTMS to three patients with severe brain injuries due to TBI. Two patients were classified as minimally conscious, while the third was in a vegetative state (55). Safety criteria included absence of contraindication to rTMS, stability of vital parameters, and more than 12 months since injury. A single session was administered to the primary motor cortex at 100% MT (20 Hz, 1000 pulses/session; 5 s train duration, 20 s inter-train interval) for 10 trains. A clinical response was not witnessed in any of the three patients, with EEG demonstrating no reaction to brain stimulation and a decrease in MT. No adverse effects or seizures were reported.

Similarly, Giovannelli and colleagues reported no clinically significant neurobehavioral change following rTMS therapy for two patients in a vegetative state due to TBI (53). Using a randomized, double-blind cross-over design with a sham stimulation control, high-frequency rTMS was applied to the left M1 at 60% stimulator output (20 Hz or sham; 1000 pulses/session) for five consecutive days. The patients did not demonstrate statistically significant improvement compared to sham stimulation as measured using the JFK Coma Recovery Scale-Revised (CRS-R) and Clinical Global Impression (CGI) rating.

### Seizure events in patients with TBI following rTMS intervention

Cavinato et al. report the occurrence of a partial and secondarily generalized tonic–clonic seizure in a 31-year-old male patient who suffered a severe TBI 8 months prior to rTMS intervention (50). MRIs following the TBI revealed diffuse hematoma in corpus callosum with mass effect over the fourth ventricle and upstream hydrocephalus. Prior to treatment, the patient received medication for gastric disorder and spasticity (2 ml Diazepam), and he had no prior personal or family history of epilepsy or seizure disorder. Treatment began once stable clinical conditions were demonstrated and baseline EEG excluded epileptiform and paroxysmal activity. Three of ten daily sessions at 90% abductor pollicis brevis MT (20 Hz, 1 s train duration, 1 min inter-train interval) applied to the DLPFC were tolerated without adverse effects. Three hours after the fourth rTMS session, the patient experienced an adversive seizure followed by a secondary generalized seizure and a complete loss of consciousness, with no urinary or bladder incontinence or tongue beating. EEG reports following the seizure showed slowing with focal spike discharges in the left fronto-central areas. Reports taken 2 days following the seizure displayed no epileptiform activity and demonstrated a return to baseline levels, with no antiepileptic medication administered at any time.

Louise-Bender Pape et al. report an electrographic seizure with no clinical accompaniment in a patient in a vegetative state following 21 sessions of rTMS (57). The patient was a 32-year-old male who had suffered a TBI 9 years prior and had a GCS score of 6 at injury. Head CT prior to treatment revealed “multicystic encephalomalacia of almost the entire right cerebral hemisphere and most of the basal ganglia as well as hypoattenuation in the anterior frontal lobe and dilatation of third and lateral ventricles.” Stimulation was applied using a paired-pulse technique with two 100 μs pulses applied at an interval of 100 ms, followed by 5 s of rest. Daily sessions of 300 pulses at 110% MT were administered over the left DLPFC. Neural activity was monitored using daily EEG recordings pre- and post-rTMS. Prior to the seizure, EEGs showed no evidence of epileptiform discharges or periodic complexes, but the authors note the presence of some neurophysiological disturbances indicated by slow wave abnormalities in the right central–parietal, right temporal, and left temporal areas. The electrographic activity originated in the right central–parietal region and extended to the mid-central and right temporal areas. The event lasted longer than 90 s, but stopped without medication. Subsequent EEGs were no different from recordings taken prior to the seizure. The patient was placed on 1000 mg levetiracetam and monitored carefully for a week. rTMS treatments were then resumed at 2% lower stimulation intensity and with 100 fewer pulses per session for an additional 19 sessions. No further adverse events were reported. The authors note that this rTMS-triggered seizure is unusual as it originated contralateral to site of stimulation.

## tDCS

### Therapeutic applications for post-injury cognitive impairments

In a double-blind cross-over study (*N* = 9), Kang et al. reported immediate but not lasting improvement in the attention of patients with TBI following a single tDCS session (59). MRI and anatomical CT scans demonstrated no significant brain atrophy or implants prior to intervention. Patients with a history of seizure or co-morbid medical/neurological conditions were excluded, and medication was kept constant throughout treatment. A single session of tDCS was administered with the anode over the left DLPFC (2 mA/25 cm^2^ × 20 min; sham 1 min on/19 min off) and the cathode over the contralateral supraorbital region. Attention was measured using a mini-mental status exam (MMSE) and computerized contrast reaction time task (CCRTT). The treatment group demonstrated improved attention from baseline compared to sham at 1-h follow-up but no significant differences were observed at 3 and 24 h post-treatment.

In a randomized double-blind controlled pilot study, Leśniak et al. found that repeated applications of tDCS in addition to daily rehabilitative cognitive training did not enhance attention or memory in patients with severe TBI (60). The study consisted of patients (*N* = 23) aged 18–45 who had experienced a TBI a minimum of 4 months prior to the study accompanied by loss of consciousness and/or post-traumatic amnesia, and no prior history of neurological/psychiatric conditions, post-injury seizures, or skull fractures/implants in the area of stimulation. Sham and treatment groups were randomized and matched by age, time since TBI, and severity of symptoms. Fifteen sessions of tDCS with the anode over the left DLPFC (1 mA/35 cm^2^ × 10 min, decreased intensity at start and finish; sham 25 s on/9.75 min off) and the cathode over the right supraorbital area were administered. Treatment was reported to be well tolerated with minor side effects. Fifteen computerized cognitive training sessions to improve memory were administered following tDCS intervention. Large effect sizes for treatment and sham group were reported but no statistically significant difference between the two groups was found.

Ulam et al. used a randomized, double-blind design to test the effect of repeated sessions of anodal tDCS on EEG oscillations and neuropsychological tests (attention, working memory, inhibitory control, cognitive flexibility, immediate and delayed memory for verbal and visual-spatial material, and emotion recognition) in patients with TBI (63). 23 patients (13 active tDCS and 13 sham) received anodal tDCS to the left PFC (1 mA/25 cm^2^ × 20 min) with the cathode over the right supraorbital area. EEG immediately after one session of tDCS demonstrated increased cortical excitability at the location of the anode. This increased excitability continued to be present 1 day following the 10th session, but was no longer restricted to the anodal location. There was no significant difference between the active and sham groups in their performance change on the neuropsychological tests, though both groups showed an overall improvement. However, there was a significant correlation between change in cortical excitability and neuropsychological improvement in the active group but not the sham group. Additionally, individuals in the active group who showed a greater slowing in EEG prior to tDCS intervention improved on a greater number of neuropsychological tests than did the rest of the active tDCS group.

### Rehabilitative applications for post-injury motor impairments

Middleton et al. report the use of tDCS in conjunction with upper extremity physical therapy for patients with motor impairments resulting for stroke and/or TBI (*N* = 5, one with TBI, one with TBI + stroke) (61). Bihemispheric tDCS was delivered with the anode over the ipsilesional motor cortex (1.5 mA/25 cm^2^ × 15 min) and cathode over the contralesional motor cortex, three times a week for 24 sessions, during strengthening and functional activities. Both TBI patients showed improvement on the Fugl-Meyer Assessment of Sensorimotor Impairment UE section (UE-FM), which assesses reflexes, range of motion, pain, light touch sensation, proprioception, movements in and out of synergy, grasp, and coordination. This improvement persisted at a 6-month follow-up. Performance changes in robotic reach and object-hit tasks were less consistent. No adverse effects of tDCS were reported. This small proof-of-concept study showed that tDCS was well tolerated by patients and can be incorporated into physical training without being a hindrance.

### Rehabilitative applications for post-injury loss of consciousness

Angelakis et al. report an attempt to restore consciousness through the use of tDCS in patients with persistent unresponsive wakefulness syndrome (UWS) following a severe TBI (58). Five daily sessions of tDCS were administered with the anode over the left DLPFC or left primary sensorimotor cortex (1 mA/35 cm^2^ × 20 min) and the cathode over the right orbital region to participants (*N* = 10, four with TBI, open head TBI excluded) with varying degrees of consciousness (assessed by JFK Coma Recovery Scale-Revised, JFK CRS-R; monitored by EEG and fMRI/FDG-PET). A 22-year-old male patient with TBI sustained 6 months prior to treatment (baseline CRS-R: 9) showed immediate improvement after a week of (CRS-R: 17) and continued improvement after second participation 3 months later (CRS-R: 19). A 19-year-old female patient with a TBI sustained 6 years prior (baseline CRS-R: 8) to intervention demonstrated no immediate response to tDCS but improved at a 1-year follow-up (CSR-R: 9). Two patients (CRS-R: 6 and 9, respectively) did not show any immediate effects (no change in CRS-R) or changes at a 1-year follow-up. Both patients were males (aged 35 and 37) with severe TBIs sustained 7 and 10 years prior, respectively. Based on the response of all patients in the study (not just those with TBI), the authors observed that the efficacy of tDCS intervention seemed to be dependent on the severity of the disordered conscious, with MCS patients responding better than UWS, and the time since injury, with more recent injuries faring better.

Thibaut et al. performed a sham-controlled randomized double-blind study to determine the effect of tDCS on consciousness in patients with UWS (*N* = 25; 6 post-traumatic) and patients in a minimally conscious state (MCS; *N* = 30; 19 post-traumatic) (62). The study employed a cross-over design in which each patient received one session of both sham and real tDCS in a randomized order. The anodal electrode was placed over the left dlPFC with the cathodal (reference) electrode over the right suborbital region (2 mA/35 cm^2^ × 20 min). A subset of patients showed a transient improvement in CRS-R score following a single session of tDCS. A group effect was seen for the MCS but not for the UWS patient group. Although the authors did not assess the effect of treatment specifically for post-traumatic patients, their presented data show that treatment had no significant effect for the 6 post-traumatic UWS patients but produced some improvement for 5 of the 19 post-traumatic MCS patients. No adverse tDCS-related side effects were observed.

## Discussion

### rTMS in the treatment of TBI

The reviewed reports reveal that rTMS can be effective in reducing neurological and psychiatric symptoms that arise as a result of a TBI. Improvements were documented in single-case reports for patients presenting with hemispatial neglect, tinnitus, auditory hallucinations, depression, and executive dysfunction. A larger-group study found that the majority of patients showed reduced post-concussion symptoms following rTMS intervention, though a sham control group was not provided. In the studies that completed follow-up examinations, some improvements were shown to persist, in varying degrees, in the weeks or months post-treatment. In this regard, rTMS demonstrates a similar ability to produce sustained symptom relief in post-TBI sequelae as in comparable non-trauma induced symptoms. Results were less encouraging with regards to the use of rTMS in states of altered consciousness. One study reported rTMS-induced neurobehavioral changes and improved consciousness, but additional attempts to recreate these results were unsuccessful. Results were also inconclusive with regards to the ability of rTMS to reduce suicidal ideation.

The inherent variability in the location and extent of brain injuries means that the symptoms and treatment requirements in each case will be different. Indeed, the reviewed reports showed a great variety in the severity and location of injuries, as well as the protocols and stimulation parameters used in their treatment. The versatility of rTMS makes it an excellent match for such a varied disorder, as the therapeutic use of the technique can be adapted to each individual case. However, this same variability makes it difficult to broadly determine its efficacy as a treatment. It is likely that there will never be a single and ideal protocol for the use of rTMS in TBI. Instead, its use will be most effective if tailored to the specific requirements of each patient or to a common set of symptoms.

The main concern with regards to the use of rTMS in cases of TBI is safety. TBI has generally been considered a contraindication for rTMS due to its association with an increased overall neural excitability and subsequent seizure risk. Indeed, patients with severe TBI do show an increased risk of unprovoked seizures, as shown by a standardized incidence ratio (SIR) of 17.0 compared to the normal population (65, 66). For this reason, care should be taken when considering rTMS treatment for this patient group. Nielson et al. suggest that brain scans and neurosurgical reports should be consulted prior to treatment of severe TBI in order to evaluate the location and severity of cortical lesions or skull plates that could unpredictably alter the path of TMS currents through brain tissue (67). In addition, they recommend the use of neuronavigational software to avoid direct stimulation of these potential hazards, as well as computational modeling of currents if stimulation is being applied close to a lesion or plate (67).

There is a strong connection between the severity of a brain injury and the subsequent risk of seizures; individuals with mild to moderate TBI have a substantially lower seizure risk than those with severe TBI. Several studies have found that any increase in the risk of seizures following a mild TBI is either marginal or non-existent compared to the general population (68, 69). It should be noted that Ferguson et al. did find a significant increase in seizure occurrence for individuals who were hospitalized with mild TBI (70). However, the authors point out that since 87.5% of people who attend an emergency room with a mild TBI are not hospitalized, their results may be skewed toward more ‘severe’ cases of mild TBI (70, 71). These findings suggest that rTMS treatment may not pose a significantly greater health risk in cases of mild TBI than in the general population. Nonetheless, the fact that mild TBI could present even a marginal increase in seizure risk is enough to warrant extra precautions.

The reviewed studies reveal that a number of methods are already being employed to help reduce the chance of an adverse event. Chiefly, EEG recordings have been used to monitor brain activity for any evidence of epileptiform discharge prior to, during, and/or following treatment. This technique is invaluable insofar as its ability to provide clinicians with real-time brain activity readings during rTMS administration. Despite this obvious utility, EEG recordings at baseline or even during treatment may not be sufficient to predict a seizure. For example, in the seizure incident reported by Cavinato and colleagues (50), the patient experienced a seizure 3 h post-treatment. In this case, clinicians had taken an EEG measurement at baseline but not during treatment sessions. However, the delay between the treatment session and the onset of the seizure suggests that brain activity indicating an impending seizure may not be immediately obvious. Nonetheless, the ability to monitor brain activity for epileptiform discharge throughout treatment is valuable. Additionally, imaging techniques, such as MRI and PET, enable clinicians to screen for lesions or other trauma-induced abnormalities that may unpredictably affect rTMS currents and pose a risk to the patient’s safety.

Nielson and colleagues recently released a list of preliminary guidelines for the safe administration of rTMS to individuals with moderate to severe TBI (67). These guidelines suggest the use of low-frequency rTMS, along with the exclusion of individuals with a history of seizures, ferrous metallic implants, or medications that are known to reduce seizure threshold. The authors also recommend that physicians review patients’ neuroimaging and neuropsychiatric reports prior to treatment to ensure that rTMS can safely be administered to the target area. Similarly, Reti and colleagues have suggested the following exclusion criteria for TBI patients who are being considered for rTMS treatment for depression: severe TBI; a history of seizures; lesions, contusions, hematomas, or surgery in the area to be treated; and the presence of a brain tumor, skull fracture, or non-TBI cerebral lesion (72). The authors also recommend that rTMS treatment not be performed within the first 3 months following a TBI due to the heightened risk of spontaneous seizure in this period and the fact that neuropsychiatric symptoms may still resolve spontaneously.

Both Nielson and Reti suggest that the use of low-frequency rTMS may be preferable to high-frequency stimulation for cases of TBI (67, 72). As previously discussed, low-frequency (≤1 Hz) stimulation is thought to reduce cortical excitability (23, 24), as observed through reductions in ipsilateral motor evoked potentials (73) and decreases in regional cerebral blood flow (74, 75). Furthermore, low-frequency rTMS has been shown to reduce seizure incidence in epileptic patients (76). Importantly, there is evidence that low-frequency rTMS to the right DLPFC can be as effective as high-frequency rTMS to the left DLPFC in the treatment of major depression (77). Taken together, these factors suggest that low-frequency rTMS may well be a superior choice for the treatment of depression in TBI patients given the higher likelihood of increased cortical excitability in this population. However, limiting TBI patients to low-frequency stimulation protocols may be unduly restrictive, especially given the low seizure risk in individuals with mild TBI and the fact that high-frequency stimulation may be the preferred treatment option for select TBI-related symptoms. For example, in a recent review of rTMS as a potential treatment for co-occurring alcohol abuse disorder, TBI, and post-traumatic stress disorder, Herrold and colleagues suggest that high-frequency, supra-threshold rTMS stimulation of the right DLPFC could help promote recovery (78). Nielson and colleagues accept that protocols exceeding their preliminary guidelines may be justifiable given appropriate compensatory steps to avoid seizure and sufficient expected benefits to warrant the risk. Careful consideration will have to be taken when comparing the risk of high-frequency stimulation in cases of moderate or severe TBI with the potential benefit to the patient of successful treatment.

The reviewed reports demonstrate that rTMS can be effective in treating symptoms of TBI, particularly in cases that do not involve disorders of consciousness. However, most of the current evidence is based on single-case reports and studies that lack sham stimulation or control groups. Larger-group, blinded, randomized, and controlled studies are necessary before firm conclusions can be drawn. The dramatic difference in seizure risk as well as type and severity of symptoms across the continuum of mild to severe TBI cases requires that additional investigations be undertaken to differentiate the efficacy of rTMS depending on injury severity. The amount of time elapsed since injury may also play a role in the effectiveness and tolerability of rTMS treatment. The initial finding of well tolerated intervention, especially amongst mild TBI patients, prompts a possible re-examination of the general safety guidelines for rTMS which currently include TBI as an exclusion criterion. The preliminary guidelines for the safe use of rTMS for TBI by Nielson et al. provide a basis for this revision.

### tDCS in the treatment of TBI

The reviewed studies show that the use of tDCS in cases of TBI has primarily been targeted at improving cognitive impairments and altered states of consciousness. tDCS has demonstrated some potential in its ability to alter attention in patients, as shown by a temporary improvement in MMSE score and reaction time following a single stimulation session (59). Kang et al. proposed that repeated sessions of longer duration or higher intensity could produce longer-lasting effects. However, two later studies found no significant benefit to a wide range of cognitive abilities – including attention, memory, and cognitive flexibility – ­following repeated applications of tDCS compared to sham (60, 63). Given that Lésniak et al. specifically recruited patients who had suffered a severe TBI, it is possible that the severity of injuries alters the efficacy of tDCS intervention. As may be the case with rTMS, the efficacy of tDCS may vary depending on both severity and time since injury.

Transcranial Direct Current Stimulation has thus far demonstrated a limited ability to improve altered states of consciousness. Roughly a quarter of TBI patients exhibiting a minimally conscious state showed a minor response to tDCS, whereas only one of nine patients with UWS showed a definite benefit from intervention (58, 62). Thibaut and colleagues suggest that the patient’s initial level of consciousness prior to intervention may be indicative of the efficacy of tDCS in such cases, with higher initial consciousness ratings faring better (62). With limited data on which to draw conclusions, further investigation will be required to ascertain the true efficacy of tDCS for TBI symptoms.

One major benefit of tDCS with this patient population is its relative safety, as tDCS does not confer the same seizure risk as rTMS (44, 59). Indeed, no major adverse effects were reported in any of the reviewed studies. In addition to the improvement of chronic symptoms post-TBI, it has been suggested that tDCS may also be beneficial in preventing brain damage during acute stages of injury by reducing glutamatergic hyperexcitability (79). Based on their observation that greater pre-treatment slowing in EEG is associated with more extensive neuropsychological improvements following tDCS, Ulam et al. suggest that EEG recording could be used as a potential biological marker of a positive response to tDCS treatment in the acute phase of TBI (63). This assertion is reinforced by their reported correlation between change in cortical excitability and neuropsychological improvement in the active tDCS group. Non-invasive brain stimulation may prove to be an important tool for increasing the efficacy of other recovery techniques by modulating neural plasticity after an acquired brain injury (80).

## Conclusion

Preliminary examinations of studies suggest that non-invasive brain stimulation techniques show potential in the treatment of TBI-related symptoms. While there is not yet enough data to draw conclusions as to the definite efficacy of rTMS or tDCS in TBI, the reviewed studies provide preliminary evidence that these methods can produce positive results in certain cases and that treatment is generally well tolerated. Larger blinded, randomized, controlled trials matched for age/sex, time since injury, and severity of symptoms must be conducted before firm conclusions can be drawn regarding their efficacy and safety. In the case of rTMS, patients would benefit from further improvement of guidelines to ensure safe protocols are followed in order to minimize the risk of adverse effects, such as seizure and syncope.

## Conflict of Interest Statement

The authors declare that the research was conducted in the absence of any commercial or financial relationships that could be construed as a potential conflict of interest.

## References

[1] Parikh S Koch M Narayan RK Traumatic brain injury. Int Anesthesiol Clin (2007) 45(3):119–35. 10.1097/AIA.0b013e318078cfe7 17622833

[2] Sorenson SB Kraus JF Occurrence, severity, and outcomes of brain injury. J Head Trauma Rehabil (1991) 6(2):1–10. 10.1097/00001199-199106000-00003

[3] McAllister TW Arciniegas D. Evaluation and treatment of postconcussive symptoms. NeuroRehabilitation (2002) 17(4):265–83. 12547976

[4] Busch CR Alpern HP. Depression after mild traumatic brain injury: a review of current research. Neuropsychol Rev (1998) 8(2):95–108. 10.1023/A:1025661200911 9658412

[5] Folmer RL Griest SE. Chronic tinnitus resulting from head or neck injuries. Laryngoscope (2003) 113(5):821–7. 10.1097/00005537-200305000-00010 12792317

[6] Fann JR Hart T Schomer KG. Treatment for depression after traumatic brain injury: a systematic review. J Neurotrauma (2009) 26(12):2383–402. 10.1089/neu.2009.1091 19698070 PMC2864457

[7] Silver JM McAllister TW Arciniegas DB. Depression and cognitive complaints following mild traumatic brain injury. Am J Psychiatry (2009) 166(6):653–61. 10.1176/appi.ajp.2009.08111676 19487401

[8] Alderfer BS Arciniegas DB Silver JM. Treatment of depression following traumatic brain injury. J Head Trauma Rehabil (2005) 20(6):544–62. 10.1097/00001199-200511000-00006 16304490

[9] De Ridder D Langguth B Posttraumatic tinnitus. In: Møller AR, editor. Textbook of Tinnitus. New York, London: Springer (2011). p. 511–6.

[10] Louise-Bender Pape T Rosenow J Lewis G Ahmed G Walker M Guernon A Repetitive transcranial magnetic stimulation-associated neurobehavioral gains during coma recovery. Brain Stimul (2009) 2(1):22–35. 10.1016/j.brs.2008.09.004 20633400

[11] Fann JR Uomoto JM Katon WJ. Sertraline in the treatment of major depression following mild traumatic brain injury. J Neuropsychiatry Clin Neurosci (2000) 12:226–32. 10.1176/jnp.12.2.226 11001601

[12] Lee H Kim SW Kim JM Shin IS Yang SJ Yoon JS. Comparing effects of methylphenidate, sertraline and placebo on neuropsychiatric sequelae in patients with traumatic brain injury. Hum Psychopharmacol (2005) 20(2):97–104. 10.1002/hup.668 15641125

[13] Rapoport MJ Chan F Lanctot K Herrmann N McCullagh S Feinstein A. An open-label study of citalopram for major depression following traumatic brain injury. J Psychopharmacol (2008) 22:860–4. 10.1177/0269881107083845 18208921

[14] Kanetani K Kimura M Endo S. Therapeutic effects of milnacipran (serotonin noradrenaline reuptake inhibitor) on depression following mild and moderate traumatic brain injury. J Nippon Med Sch (2003) 70:313–20. 10.1272/jnms.70.313 12928711

[15] Kaelin DL Cifu DX Matthies B. Methylphenidate effect on attention deficit in the acutely brain-injured adult. Arch Phys Med Rehabil (1996) 77(1):6–9. 10.1016/S0003-9993(96)90211-7 8554476

[16] Plenger PM Dixon CE Castillo RM Frankowski RF Yablon SA Levin HS. Subacute methylphenidate treatment for moderate to moderately severe traumatic brain injury: a preliminary double-blind placebo-controlled study. Arch Phys Med Rehabil (1996) 77(6):536–40. 10.1016/S0003-9993(96)90291-9 8831468

[17] Ashman TA Cantor JB Gordon WA Spielman L Flanagan S Ginsberg A A randomized controlled trial of sertraline for the treatment of depression in persons with traumatic brain injury. Arch Phys Med Rehabil (2009) 90(5):733–40. 10.1016/j.apmr.2008.11.005 19406291

[18] Arciniegas DB Anderson CA Topkoff J McAllister TW. Mild traumatic brain injury: a neuropsychiatric approach to diagnosis, evaluation, and treatment. Neuropsychiatr Dis Treat (2005) 1(4):311–27. 18568112 PMC2424119

[19] Kreuzer PM Landgrebe M Frank E Langguth B. Repetitive transcranial magnetic stimulation for the treatment of chronic tinnitus after traumatic brain injury: a case study. J Head Trauma Rehabil (2013) 28(5):386–9. 10.1097/HTR.0b013e318254736e 22688213

[20] Cosentino G Giglia G Palermo A Panetta ML Lo Baido R Brighina F A case of post-traumatic complex auditory hallucinosis treated with rTMS. Neurocase (2010) 16(3):267–72. 10.1080/13554790903456191 20104391

[21] Hegel MT Martin JB. Behavioral treatment of pulsatile tinnitus and headache following traumatic head injury. Objective polygraphic assessment of change. Behav Modif (1998) 22(4):563–72. 10.1177/01454455980224007 9755652

[22] Demirtas-Tatlidede A Vahabzadeh-Hagh AM Bernabeu M Tormos JM Pascual-Leone A. Noninvasive brain stimulation in traumatic brain injury. J Head Trauma Rehabil (2012) 27(4):274–92. 10.1097/HTR.0b013e318217df55 21691215 PMC3342413

[23] Rossi S Hallett M Rossini PM Pascual-Leone A Safety, ethical considerations, and application guidelines for the use of transcranial magnetic stimulation in clinical practice and research. Clin Neurophysiol (2009) 120(12):2008–39. 10.1016/j.clinph.2009.08.016 19833552 PMC3260536

[24] Wassermann EM Grafman J Berry C Hollnagel C Wild K Clark K Use and safety of a new repetitive transcranial magnetic stimulator. Electroencephalogr Clin Neurophysiol (1996) 101(5):412–7. 10.1016/0924-980X(96)96004-X 8913194

[25] Figiel GS Epstein C McDonald WM Amazon-Leece J Figiel L Saldivia A The use of rapid-rate transcranial magnetic stimulation (rTMS) in refractory depressed patients. J Neuropsychiatry Clin Neurosci (1998) 10(1):20–5. 10.1176/jnp.10.1.20 9547462

[26] O’Reardon JP Solvason HB Janicak PG Sampson S Isenberg KE Nahas Z Efficacy and safety of transcranial magnetic stimulation in the acute treatment of major depression: a multisite randomized controlled trial. Biol Psychiatry (2007) 62(11):1208–16. 10.1016/j.biopsych.2007.01.018 17573044

[27] George MS Lisanby SH Avery D McDonald WM Durkalski V Pavlicova M Daily left prefrontal transcranial magnetic stimulation therapy for major depressive disorder: a sham-controlled randomized trial. Arch Gen Psychiatry (2010) 67(5):507–16. 10.1001/archgenpsychiatry.2010.46 20439832

[28] Castel-Lacanal E Tarri M Loubinoux I Gasq D de Boissezon X Marque P Transcranial magnetic stimulation in brain injury. Ann Fr Anesth Reanim (2014) 33(2):83–7. 10.1016/j.annfar.2013.11.006 24378049

[29] Hsu WY Cheng CH Liao KK Lee IH Lin YY. Effects of repetitive transcranial magnetic stimulation on motor functions in patients with stroke: a meta-­analysis. Stroke (2012) 43(7):1849–57. 10.1161/STROKEAHA.111.649756 22713491

[30] Barwood CH Murdoch BE Whelan BM Lloyd D Riek S O’Sullivan J The effects of low frequency repetitive transcranial magnetic stimulation (rTMS) and sham condition rTMS on behavioural language in chronic non-fluent aphasia: short term outcomes. NeuroRehabilitation (2011) 28(2):113–28. 10.3233/NRE-2011-0640 21447912

[31] Brighina F Bisiach E Oliveri M Piazza A La Bua V Daniele O 1 Hz repetitive transcranial magnetic stimulation of the unaffected hemisphere ameliorates contralesional visuospatial neglect in humans. Neurosci Lett (2003) 336(2):131–3. 10.1016/S0304-3940(02)01283-1 12499057

[32] Leo RJ Latif T. Repetitive transcranial magnetic stimulation (rTMS) in experimentally induced and chronic neuropathic pain: a review. J Pain (2007) 8(6):453–9. 10.1016/j.jpain.2007.01.009 17434804

[33] Leung A Donohue M Xu R Lee R Lefaucheur JP Khedr EM rTMS for suppressing neuropathic pain: a meta-analysis. J Pain (2009) 10(12):1205–16. 10.1016/j.jpain.2009.03.010 19464959

[34] Mori F Koch G Foti C Bernardi G Centonze D. The use of repetitive transcranial magnetic stimulation (rTMS) for the treatment of spasticity. Prog Brain Res (2009) 175:429–39. 10.1016/S0079-6123(09)17528-3 19660671

[35] Knoch D Gianotti LR Pascual-Leone A Treyer V Regard M Hohmann M Disruption of right prefrontal cortex by low-frequency repetitive transcranial magnetic stimulation induces risk-taking behavior. J Neurosci (2006) 26(24):6469–72. 10.1523/JNEUROSCI.0804-06.2006 16775134 PMC6674035

[36] Nitsche MA Paulus W. Excitability changes induced in the human motor cortex by weak transcranial direct current stimulation. J Physiol (2000) 527(Pt 3):633–9. 10.1111/j.1469-7793.2000.t01-1-00633.x 10990547 PMC2270099

[37] Nitsche MA Paulus W. Sustained excitability elevations induced by transcranial DC motor cortex stimulation in humans. Neurology (2001) 57(10):1899–901. 10.1212/WNL.57.10.1899 11723286

[38] Fregni F Boggio PS Santos MC Lima M Vieira AL Rigonatti SP Noninvasive cortical stimulation with transcranial direct current stimulation in Parkinson’s disease. Mov Disord (2006) 21(10):1693–702. 10.1002/mds.21012 16817194

[39] Boggio PS Nunes A Rigonatti SP Nitsche MA Pascual-Leone A Fregni F. Repeated sessions of noninvasive brain DC stimulation is associated with motor function improvement in stroke patients. Restor Neurol Neurosci (2007) 25(2):123–9. 17726271

[40] Nitsche MA Boggio PS Fregni F Pascual-Leone A. Treatment of depression with transcranial direct current stimulation (tDCS): a review. Exp Neurol (2009) 219(1):14–9. 10.1016/j.expneurol.2009.03.038 19348793

[41] Boggio PS Rigonatti SP Ribeiro RB Myczkowski ML Nitsche MA Pascual-Leone A A randomized, double-blind clinical trial on the efficacy of cortical direct current stimulation for the treatment of major depression. Int J Neuropsychopharmacol (2008) 11(2):249–54. 10.1017/S1461145707007833 17559710 PMC3372849

[42] Berlim MT Van den Eynde F Daskalakis ZJ. Clinical utility of transcranial direct current stimulation (tDCS) for treating major depression: a systematic review and meta-analysis of randomized, double-blind and sham-­controlled trials. J Psychiatr Res (2013) 47(1):1–7. 10.1016/j.jpsychires.2012.09.025 23084964

[43] Nitsche MA Paulus W. Noninvasive brain stimulation protocols in the treatment of epilepsy: current state and perspectives. Neurotherapeutics (2009) 6(2):244–50. 10.1016/j.nurt.2009.01.003 19332316 PMC5084200

[44] Poreisz C Boros K Antal A Paulus W. Safety aspects of transcranial direct current stimulation concerning healthy subjects and patients. Brain Res Bull (2007) 72(4–6):208–14. 10.1016/j.brainresbull.2007.01.004 17452283

[45] Nitsche MA Liebetanz D Antal A Lang N Tergau F Paulus W Modulation of cortical excitability by weak direct current stimulation – technical, safety and functional aspects. Suppl Clin Neurophysiol (2003) 56:255–76. 10.1016/S1567-424X(09)70230-2 14677403

[46] Brunoni AR Valiengo L Baccaro A Zanão TA de Oliveira JF Goulart A The sertraline vs. electrical current therapy for treating depression clinical study: results from a factorial, randomized, controlled trial. JAMA Psychiatry (2013) 70(4):383–91. 10.1001/2013.jamapsychiatry.32 23389323

[47] Monti A Cogiamanian F Marceglia S Ferrucci R Mameli F Mrakic-Sposta S Improved naming after transcranial direct current stimulation in aphasia. J Neurol Neurosurg Psychiatry (2008) 79(4):451–3. 10.1136/jnnp.2007.135277 18096677

[48] Fregni F Boggio PS Lima MC Ferreira MJ Wagner T Rigonatti SP A sham-controlled, phase II trial of transcranial direct current stimulation for the treatment of central pain in traumatic spinal cord injury. Pain (2006) 122(1–2):197–209. 10.1016/j.pain.2006.02.023 16564618

[49] Bonni S Mastropasqua C Bozzali M Caltagirone C Koch G. Theta burst stimulation improves visuo-spatial attention in a patient with traumatic brain injury. Neurol Sci (2013) 34(11):2053–6. 10.1007/s10072-013-1412-y 23532550

[50] Cavinato M Iaia V Piccione F Repeated sessions of sub-threshold 20-Hz rTMS. Potential cumulative effects in a brain-injured patient. Clin Neurophysiol (2012) 123(9):1893–5. 10.1016/j.clinph.2012.02.066 22405994

[51] Fitzgerald PB Hoy KE Maller JJ Herring S Segrave R McQueen S Transcranial magnetic stimulation for depression after a traumatic brain injury: a case study. J ECT (2011) 27(1):38–40. 10.1097/YCT.0b013e3181eb30c6 20938348

[52] George MS Raman R Benedek DM Pelic CG Grammer GG Stokes KT A two-site pilot randomized 3 day trial of high dose left prefrontal repetitive transcranial magnetic stimulation (rTMS) for suicidal inpatients. Brain Stimul (2014) 7(3):421–31. 10.1016/j.brs.2014.03.006 24731434

[53] Giovannelli F Chiaramonti R Bianco G Godone M Battista D Cardinali C Lack of behavioural effects of high-frequency rTMS in vegetative state: a randomised, double blind, sham-controlled, cross-over study. Neurophysiol Clin (2014) 125(S1):S243 10.1016/S1388-2457(14)50791-2

[54] Koski L Kolivakis T Yu C Chen JK Delaney S Ptito A. Noninvasive brain stimulation for persistent postconcussion symptoms in mild traumatic brain injury. J Neurotrauma (2015) 32(1):38–44. 10.1089/neu.2014.3449 24955920

[55] Manganotti P Formaggio E Storti SF Fiaschi A Battistin L Tonin P Effect of high-frequency repetitive transcranial magnetic stimulation on brain excitability in severely brain-injured patients in minimally conscious or vegetative state. Brain Stimul (2013) 6(6):913–21. 10.1016/j.brs.2013.06.006 23928101

[56] Pachalska M Łukowicz M Kropotov JD Herman-Sucharska I Talar J. Evaluation of differentiated neurotherapy programs for a patient after severe TBI and long term coma using event-related potentials. Med Sci Monit (2011) 17(10):CS120–8. 10.12659/MSM.881970 21959618 PMC3539468

[57] Louise-Bender Pape T Rosenow JM Patil V Steiner M Harton B Guernon A rTMS safety for two subjects with disordered consciousness after traumatic brain injury. Brain Stimul (2014) 7(4):620–2. 10.1016/j.brs.2014.03.007 24836500

[58] Angelakis E Liouta E Andreadis N Korfias S Ktonas P Stranjalis G Transcranial direct current stimulation effects in disorders of consciousness. Arch Phys Med Rehabil (2014) 95(2):283–9. 10.1016/j.apmr.2013.09.002 24035769

[59] Kang EK Kim DY Paik NJ. Transcranial direct current stimulation of the left prefrontal cortex improves attention in patients with traumatic brain injury: a pilot study. J Rehabil Med (2012) 44(4):346–50. 10.2340/16501977-0947 22434324

[60] Leśniak M Polanowska K Seniów J Członkowska A. Effects of repeated anodal tDCS coupled with cognitive training for patients with severe traumatic brain injury: a pilot randomized controlled trial. J Head Trauma Rehabil (2014) 29(3):E20–9. 10.1097/HTR.0b013e318292a4c2 23756431

[61] Middleton A Fritz SL Liuzzo DM Newman-Norlund R Herter TM. Using clinical and robotic assessment tools to examine the feasibility of pairing tDCS with upper extremity physical therapy in patients with stroke and TBI: a consideration-of-concept pilot study. NeuroRehabilitation (2014) 35(4):741–54. 10.3233/NRE-141178 25323084 PMC4268358

[62] Thibaut A Bruno MA Ledoux D Demertzi A Laureys S. tDCS in patients with disorders of consciousness: sham-controlled randomized double-blind study. Neurology (2014) 82(13):1112–8. 10.1212/WNL.0000000000000260 24574549

[63] Ulam F Shelton C Richards L Davis L Hunter B Fregni F Cumulative effects of transcranial direct current stimulation on EEG oscillations and attention/working memory during subacute neurorehabilitation of traumatic brain injury. Clin Neurophysiol (2015) 126(3):486–96. 10.1016/j.clinph.2014.05.015 24947595

[64] Koch G Bonnì S Giacobbe V Bucchi G Basile B Lupo F θ-burst stimulation of the left hemisphere accelerates recovery of hemispatial neglect. Neurology (2012) 78(1):24–30. 10.1212/WNL.0b013e31823ed08f 22170878

[65] Bazarian JJ Cernak I Noble-Haeusslein L Potolicchio S Temkin N. Long-term neurologic outcomes after traumatic brain injury. J Head Trauma Rehabil (2009) 24(6):439–51. 10.1097/HTR.0b013e3181c15600 19940677

[66] Annegers JF Hauser WA Coan SP Rocca WA. A population-based study of seizures after traumatic brain injuries. N Engl J Med (1998) 338(1):20–4. 10.1056/NEJM199801013380104 9414327

[67] Nielson DM McKnight CA Patel RN Kalnin AJ Mysiw WJ. Preliminary guidelines for safe and effective use of repetitive transcranial magnetic stimulation in moderate to severe traumatic brain injury. Arch Phys Med Rehabil (2015) 96(4 Suppl):S138–44. 10.1016/j.apmr.2014.09.010 25281871

[68] Annegers JF Grabow JD Groover RV Laws ER Elveback LR Kurland LT. Seizures after head trauma: a population study. Neurology (1980) 30(7 Pt 1):683–9. 10.1212/WNL.30.7.683 7190235

[69] Annegers JF Coan SP. The risks of epilepsy after traumatic brain injury. Seizure (2000) 9(7):453–7. 10.1053/seiz.2000.0458 11034867

[70] Ferguson PL Smith GM Wannamaker BB Thurman DJ Pickelsimer EE Selassie AW. A population-based study of risk of epilepsy after hospitalization for traumatic brain injury. Epilepsia (2010) 51(5):891–8. 10.1111/j.1528-1167.2009.02384.x 19845734

[71] Selassie AW Pickelsimer EE Frazier L Jr Ferguson PL. The effect of insurance status, race, and gender on ED disposition of persons with traumatic brain injury. Am J Emerg Med (2004) 22(6):465–73. 10.1016/j.ajem.2004.07.024 15520941

[72] Reti IM Schwarz N Bower A Tibbs M Rao V. Transcranial magnetic stimulation: a potential new treatment for depression associated with traumatic brain injury. Brain Inj (2015) 7:1–9. 10.3109/02699052.2015.1009168 25950260

[73] Fitzgerald PB Fountain S Daskalakis ZJ. A comprehensive review of the effects of rTMS on motor cortical excitability and inhibition. Clin Neurophysiol (2006) 117(12):2584–96. 10.1016/j.clinph.2006.06.712 16890483

[74] Kimbrell TA Dunn RT George MS Danielson AL Willis MW Repella JD Left prefrontal-repetitive transcranial magnetic stimulation (rTMS) and regional cerebral glucose metabolism in normal volunteers. Psychiatry Res (2002) 115(3):101–13. 10.1016/S0925-4927(02)00041-0 12208488

[75] Loo CK Sachdev PS Haindl W Wen W Mitchell PB Croker VM High (15 Hz) and low (1 Hz) frequency transcranial magnetic stimulation have different acute effects on regional cerebral blood flow in depressed patients. Psychol Med (2003) 33(6):997–1006. 10.1017/S0033291703007955 12946084

[76] Hsu WY Cheng CH Lin MW Shih YH Liao KK Lin YY. Antiepileptic effects of low frequency repetitive transcranial magnetic stimulation: a meta-analysis. Epilepsy Res (2011) 96(3):231–40. 10.1016/j.eplepsyres.2011.06.002 21715144

[77] Chen J Zhou C Wu B Wang Y Li Q Wei Y Left versus right repetitive transcranial magnetic stimulation in treating major depression: a meta-­analysis of randomised controlled trials. Psychiatry Res (2013) 210(3):1260–4. 10.1016/j.psychres.2013.09.007 24113125

[78] Herrold AA Kletzel SL Harton BC Chambers RA Jordan N Pape TL. Transcranial magnetic stimulation: potential treatment for co-occurring alcohol, traumatic brain injury and posttraumatic stress disorders. Neural Regen Res. (2014) 9(19):1712–30. 10.4103/1673-5374.143408 25422632 PMC4238159

[79] Vernet M Bashir S Enam S Kumru H Pascual-Leone A Electrophysiologic techniques. 2nd ed In: Zasler ND Katz DI Zafonte RD, editors. Brain Injury Medicine: Principles and Practice. New York, NY: Demos Medical (2013). p. 230–47.

[80] Bashir S Mizrahi I Weaver K Fregni F Pascual-Leone A. Assessment and modulation of neural plasticity in rehabilitation with transcranial magnetic stimulation. PM R (2010) 2(12 Suppl 2):S253–68. 10.1016/j.pmrj.2010.10.015 21172687 PMC3951769

